# Mitochondrial Dysfunction Increases Arrhythmic Triggers and Substrates; Potential Anti-arrhythmic Pharmacological Targets

**DOI:** 10.3389/fcvm.2021.646932

**Published:** 2021-02-15

**Authors:** Khalil Saadeh, Ibrahim Talal Fazmin

**Affiliations:** ^1^School of Clinical Medicine, University of Cambridge, Cambridge, United Kingdom; ^2^Faculty of Health and Medical Sciences, University of Surrey, Guildford, United Kingdom; ^3^Royal Papworth Hospital NHS Foundation Trust, Cambridge, United Kingdom

**Keywords:** arrhythmias, mitochondrial dysfunction, ROS, aging, ion channels

## Abstract

Incidence of cardiac arrhythmias increases significantly with age. In order to effectively stratify arrhythmic risk in the aging population it is crucial to elucidate the relevant underlying molecular mechanisms. The changes underlying age-related electrophysiological disruption appear to be closely associated with mitochondrial dysfunction. Thus, the present review examines the mechanisms by which age-related mitochondrial dysfunction promotes arrhythmic triggers and substrate. Namely, via alterations in plasmalemmal ionic currents (both sodium and potassium), gap junctions, cellular Ca^2+^ homeostasis, and cardiac fibrosis. Stratification of patients' mitochondrial function status permits application of appropriate anti-arrhythmic therapies. Here, we discuss novel potential anti-arrhythmic pharmacological interventions that specifically target upstream mitochondrial function and hence ameliorates the need for therapies targeting downstream changes which have constituted traditional antiarrhythmic therapy.

## Introduction

Aging is the progressive decline in the fitness of an organism due to cumulative organ-specific physiological deterioration (1, 2). The advancement of modern medicine is thus reflected in increasing human life expectancy (3). However, an aging population offers novel medical challenges with increasing prevalence of a number of conditions including cardiovascular, oncological, and neurological diseases. The incidence of cardiovascular diseases increases exponentially in the elderly population (4, 5). In the aging population, cardiovascular diseases are the leading cause of morbidity and mortality (3, 5, 6). Thus, cardiovascular diseases have a prevalence of 82.6 million (36.2%) in the United States (4) carrying a greater financial burden than any other group of diseases including cancer and benign neoplasms (4). In 2007, 33.6% of all deaths (~814,000 people) in the United States had cardiovascular disease as the underlying cause of death (4). It is estimated that eliminating mortality from cardiovascular diseases would add between 5.5 and 7 years to mean life expectancy (4, 7). As the aging population continues to increase, with the number of elderly people predicted to double in the next 25 years in the United States age related cardiovascular diseases will continue to represent a major public health concern (5, 8). As such, it is increasingly important to be able to stratify risk of cardiovascular diseases by age and understand their underlying age-related molecular mechanisms in order to develop effective pharmacological therapies.

Within cardiovascular diseases, cardiac arrhythmias arise due to disruption in the orderly sequence of cardiomyocyte action potential activation and recovery through successive regions of the myocardium compromising cardiac function (9, 10). Of atrial arrhythmias, atrial fibrillation (AF) is the most common type. It is associated with major morbidity by increasing the risk of stroke and heart failure, as well as all-cause mortality (11–13). Ventricular arrhythmias such as ventricular tachycardia often degenerating into ventricular fibrillation (VF) are also a major public health concern. They constitute the primary cause of sudden cardiac death (SCD), which accounts for 4–5 million deaths/year worldwide (14) representing over 5% of overall mortality (15).

Incidence of cardiac rhythm abnormalities increases exponentially with age (6, 16, 17). Hence, incidence of AF in the general population increases 23-fold from the 20–24 to the 55–59 years age group (18, 19) and reaches a prevalence of over 13% in the >80 years age group (20). Similarly, incidence of VF in the general population increases 18-fold from the 20–24 to the 55–59 years age group (21).

Primary electrical abnormalities due to congenital channelopathies represent an important cause of arrhythmias and SCD (15, 22, 23). These include long QT syndrome 3 (LQT3) arising from a gain-of-function mutation in the cardiac sodium Na_V_1.5 channel gene SCN5A, Brugada Syndrome (BrS) arising from a loss-of-function mutation in the SCN5A gene, and catecholaminergic polymorphic ventricular tachycardia (CPVT) arising from a gain-of-function mutation in RyR2 gene or loss-of-function mutation in CASQ2 gene encoding cardiac calcium homeostasis proteins (23, 24). Proarrhythmic inherited channelopathies demonstrate how each component of the cardiac electrophysiological system contributes to arrhythmogenesis. Thus, studying those channelopathies has been crucial to elucidating the mechanisms underlying arrhythmogenesis in the general and aging population.

Interestingly, arrhythmic risk in individuals with many inherited channelopathies, such as BrS and LQT3, increases markedly with age, despite these individuals carrying the proarrhythmic mutation from birth (25). For example, LQT3 patients show significantly increased arrhythmic risk after the 40 years of age (26, 27). In CPVT however, patients are usually diagnosed in the first or second decade of life with the mean age of onset of symptoms, usually a syncopal episode, is between age seven and 12 years (28). Therefore, select channelopathies demonstrate an excellent paradigm to study the effects of age-related molecular changes on susceptible hearts with inherent proarrhythmic tendency. This will elucidate the molecular mechanisms underlying proarrhythmic changes with age and hence offer novel anti-arrhythmic pharmacological targets.

## Aging and Energetic Dysfunction

It has long been established that central to the aging process of any organ is energetic dysfunction giving rise to free radical reactive oxygen species (ROS) that cause damage to cellular macromolecules, accumulation of this damage leads to the physiological compromise seen in aging (5, 29). Current evidence suggests that mitochondrial dysregulation is the cause and primary target of energetic dysfunction and free radical production (5, 30). Thus, transgenic mice overexpressing the cellular antioxidant catalase targeted to the mitochondria had a reduced ROS-induced damage of the mitochondria and significantly increased lifespan (31).

A clear link exists between aging and mitochondrial dysfunction, occurring through various mechanism which include mitochondrial DNA damage, clonal expansion of deleterious mutations in mitochondrial DNA and deficiencies in the enzymes of the mitochondrial respiratory chain, such as cytochrome-c-oxidase (32–36). This phenomenon of aging driving mitochondrial genetic instability has thus been observed not just in humans but several other mammalian species, including in mice, rats, and rhesus monkeys (37–39). The link between aging and mitochondrial dysfunction appears to be bidirectional. For example, increased levels of mitochondrial DNA mutations are associated with a premature aging syndrome in mice (34, 40). Therefore, it is apparent that understanding the biology of mitochondrial instability via mitochondrial DNA mutations and enzyme deficiencies is key to understanding cellular- and tissue-level changes that underlie aging-related pathology.

As such, damaged and dysfunctional mitochondria result in production of high levels of ROS, disrupted mitochondrial membrane potentials, reduced ATP production capacity, and altered cellular redox potential (5, 41–44). The consequent aberrant mitochondrial signaling predisposes the myocardium to arrhythmias (9, 43).

This is demonstrated clinically and experimentally. Mitochondria from human AF patients are abnormal in terms of morphology and function and show DNA damage (45–48). Abnormal mitochondria are also seen in animal models of AF and ventricular arrhythmia (49–51). Additionally, inherited errors of metabolism involving mitochondria such as Kearns-Sayre syndrome manifest symptomatically as fatal rhythm abnormalities (52). Detailed electrophysiological studies in peroxisome proliferator-activated receptor gamma coactivator 1-alpha (Pgc-1α) and Pgc-1β knockout models of mitochondrial dysfunction yield similar overt arrhythmic phenotypes whilst also yielding information on the ionic basis of these arrhythmias. For example, Pgc-1β-/- mice show decreased atrial and ventricular conduction velocity, which may be attributed to reduced voltage gated inward Na^+^ currents (53–60).

The present review separates the pro-arrhythmic molecular changes in aging into multiple pathways. However, this is largely to make the topic more accessible and easier to conceptualize. In reality these pathways are dependent upon and interact with each other through complex feedback loops. Physiological interactions which are important to the arrhythmic process are also highlighted.

## Mice Models

Animal models have been pivotal in studying arrhythmias, permitting experimentation on the cellular and system level. Mice, often with electrophysiologically stable 129/Sv or C57BL/6 genetic backgrounds, have thus far represented the main transgenic system for modeling arrhythmic syndromes (61, 62) typically via well-defined mutations strategically positioned to reflect the genotypes associated with these syndromes and reliably reflecting their phenotype (9, 23, 63). From a practical aspect, mice are inexpensive, easily maintained, and reproduce rapidly thus allowing provision of aged mice over relatively short periods (25). Mice also reflect the human aging process such that they complete their growth before reproduction commences (1, 64). Furthermore, confounding risk factors which influence cardiovascular health (e.g., smoking and hypercholesterolaemia) are absent in murine models and as such their hearts reflect intrinsic cardiac aging (1). Together, these features of murine models make them valuable in the study of the mechanisms underlying cardiac aging.

Despite differences with regards to heart rate, heart size, as well as calcium- and potassium-mediated repolarization currents, which limits their ability to model conditions such as LQT1 and LQT2, murine and human hearts show significant structural and physiological resemblances (65). Structural similarities include similar conducting, sinoatrial and atrioventricular nodes, His-Purkinje systems and contracting atrial and ventricular chambers (25, 65, 66). Important electrophysiological similarities exist especially with respect to their action potential (AP) waveforms where they both share the same role of the inward sodium current in mediating phase 0 depolarization (25, 65) as well as similar transmural differences in AP duration and AP conduction velocities (65, 67, 68). These similarities are critical in permitting mice to effectively model LQT3 and BrS (9).

## Mitochondrial Dysfunction and Disrupted Surface Membrane Ionic Currents

### Mitochondrial Dysfunction and Sodium Currents

**Figure 1:**
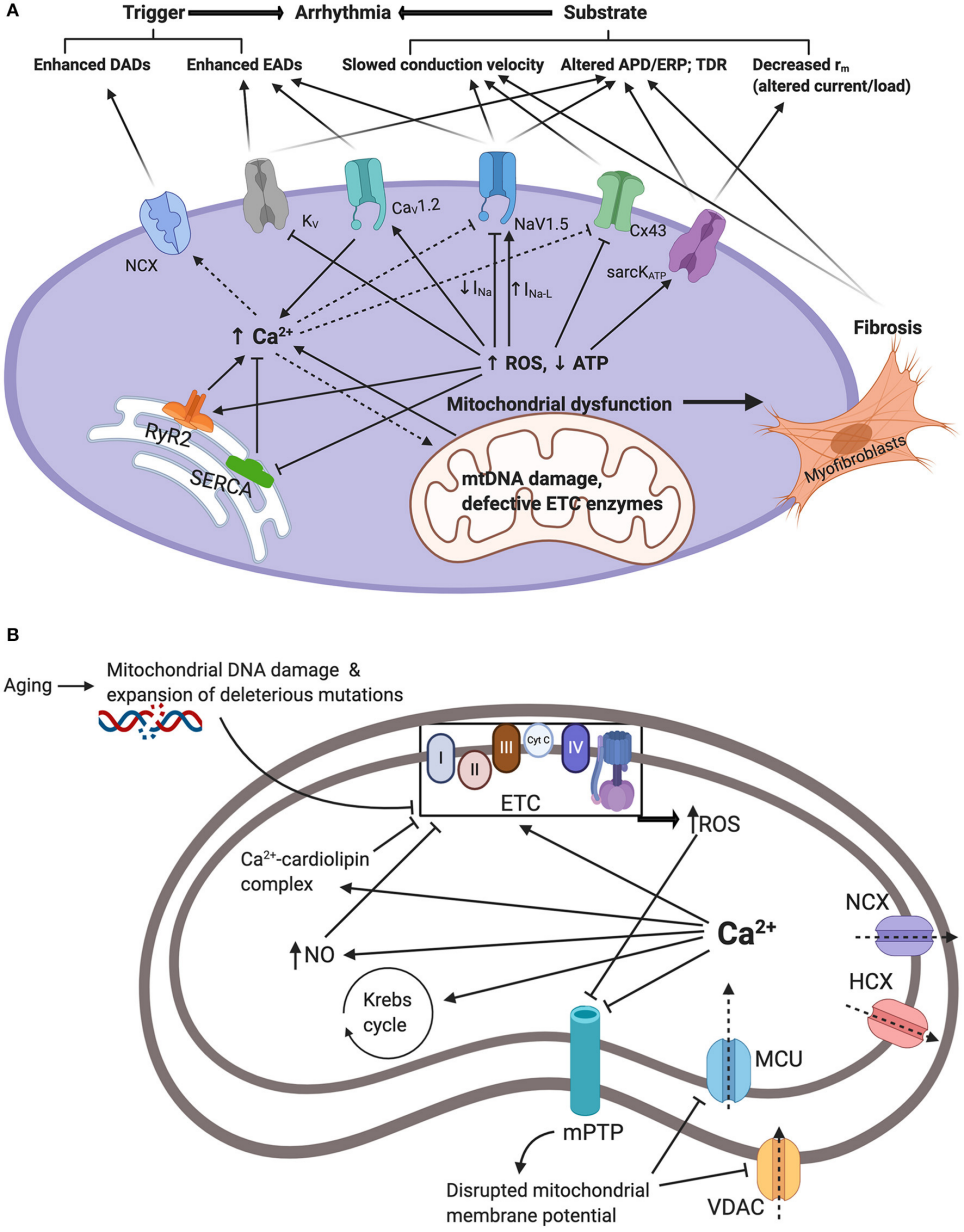
**(A)** Summarizes the interacting mechanisms by which mitochondrial dysfunction promotes arrhythmogenesis. Mitochondrial dysfunction driven by mitochondrial DNA damage and defective electron transport chain (ETC) enzymes results in reduced ATP and increased reactive oxygen species (ROS) production. In turn, this modifies function and/or expression of sarcolemmal ATP-sensitive K^+^ channels (sarcK_ATP_), gap junction proteins (Cx43), cardiac sodium channels (Na_V_1.5), cardiac L-type voltage gated Ca^2+^ channels (Ca_V_1.2), and cardiac voltage gated potassium channels (K_V_). Moreover, ROS modify endoplasmic reticulum Ca^2+^ homeostasis proteins ryanodine receptor (RyR) and sarco/endoplasmic reticulum Ca^2+^-ATPase (SERCA) resulting in elevated cellular Ca^2+^ levels which drives the depolarizing Na^+^-Ca^2+^ exchanger (NCX). Together, these changes increase arrhythmic triggers and substrates. APD/ERP: action potential duration/effective refractory period ratios; DADs: delayed after-depolarizations; EADs: early after-depolarizations; TDR: transmural dispersion of repolarization. **(B)** Summarizes mitochondrial Ca^2+^ handling and its relation to mitochondrial dysfunction and elevated cytosolic Ca^2+^. Ca^2+^ enters the mitochondria via voltage dependent anion channels (VDAC) and mitochondrial calcium uniporter (MCU). This is driven by the negative mitochondrial membrane potential (~−180mV). Increased cytosolic Ca^2+^ through mechanisms demonstrated in **(A)** will result in increased uptake and concentration of mitochondrial Ca^2+^. Mitochondrial Ca^2+^ overload contributes to mitochondrial dysfunction. Firstly, Ca^2+^ stimulation of the Krebs cycle and the oxidative phosphorylation electron transport chain increasing electron leakage and ROS by-products. Secondly, Ca^2+^-cardiolipin complexation disrupting mitochondrial lipid and protein arrangement causing proteins such as cytochrome c dislocation and inhibition of the electron transport chain and ROS production. Thirdly, through activation of nitric oxide synthase generating NO radicals which inhibit components of the ETC and promote ROS production. Increased ROS and cytosolic Ca^2+^ also inhibit mitochondrial permeability transition pores (mPTP) impairing Ca^2+^ uptake and contributing to increased cytosolic Ca^2+^. Ca^2+^ is also extruded through the hydrogen-calcium exchanger (HCX) and the mitochondrial sodium-calcium exchanger (NCX).

ROS promote both arrhythmic triggers and substrates and hence exert numerous proarrhythmic actions through modulation of intracellular and cell surface ion channels. Firstly, ROS modifies the expression and function of voltage gated Na^+^ carrying channel, Na_V_1.5, causing a decrease in the fast depolarizing component of the sodium current (I_Na_) but an increase in the late sodium current (I_Na−L_) (9, 69–72). Thus, in human embryonic kidney (HEK) cells and C57BL/6 murine cardiomyocytes, application of cytosolic NADH and mitochondrial ROS-generating molecules, such as the complex III inhibitor Anti-mycin A, reduced I_Na_ (69, 70). However, this effect was blocked by application of mitoTEMPO a specific scavenger of mitochondrial superoxide (69, 70). In murine hearts modeling mitochondrial dysfunction, increased age and Pgc-1β-/- genotype interacted to decrease atrial Na_V_1.5 channel expression (36). Furthermore, the A280V mutation in glycerol-3-phosphate dehydrogenase 1-like (GPD1-L) protein, which causes Brugada syndrome, reduces I_Na_ via increasing cytosolic NADH and mitochondrial ROS levels (73, 74). Additionally, through oxidation of the Ca^2+^-/calmodulin-dependent kinase II (CaMKII), ROS has also been shown to enhance I_Na−L_ (75–78). Together, these alterations in the cardiomyocyte sodium current promote arrhythmogenesis through increased triggered activity and arrhythmic substrate. These findings are summarized in Figure 1.

Increased I_Na−L_ prolongs membrane repolarization and as such allows the development of early-after depolarizations (EADs) through reactivation of voltage-gated Ca^2+^ channels (VGCC), and in turn, EADs can trigger arrhythmic events (79, 80). In addition, repolarization defects caused by increased I_Na−L_ promote spatiotemporal heterogeneity and transmural dispersion of repolarization arrhythmic substrate (79, 80). The changes in I_Na_ have profound consequences on ordered action potential propagation through the myocardium. Cardiac conduction velocity is largely determined by the maximum rate of membrane depolarization (dV/dt)_max_, which in turn is determined by I_Na_ and conducted by the Na_V_1.5 channel. Reduced conduction velocity forms the arrhythmic substrate associated with re-entrant arrhythmias (81, 82). Interestingly, these findings may explain the change in phenotype with age in certain channelopathies. For example, an overlap syndrome in aging LQT3 patients describes the emergence of Brugada syndrome patterns on surface ECGs in addition to the prolonged QT interval indicative of LQT3 (83, 84). Similarly, electrophysiological studies on murine LQT3 models report decreased conduction velocity in aged, but not young, hearts (85, 86). Therefore, age-related mitochondrial dysfunction and ROS generation may account for the activation abnormalities that appear later in life in LQT3 patients and associated with increased arrhythmic risk. Another important consideration is the close link between intracellular Na^+^ and Ca^2+^ regulation. Hence, the increase in I_Na−L_ causing increased [Na^+^]_i_ has been shown to increase [Ca^2+^]_i_ largely through reversing the activity of the sodium-calcium exchanger (NCX) (87, 88). In turn, as discussed later, elevated [Ca^2+^]_i_ promotes proarrhythmic electrophysiological changes including inhibition of I_Na_ (89, 90).

### Mitochondrial Dysfunction and Gap Junctions

Cardiac conduction velocity is also influenced by the axial resistance (r_a_) to local current flow between cells as determined by intercellular gap junction channels formed by connexin (Cx) proteins (82, 91). ACE8/8 mice are produced by placing the angiotensin-converting enzyme (ACE) gene under the control of the α-myosin heavy chain promoter using targeted homologous recombination. This results in significantly increased cardiac ACE and angiotensin II levels. Studies on ACE8/8 mice demonstrated that increased ROS production through renin-angiotensin system (RAS) activation, increased expression and activation of the redox-sensitive tyrosine kinase cSrc in ventricular cardiomyocytes resulting in reduced Cx43 function and expression (74, 92, 93). This reduced conduction velocity and increased risk of ventricular arrhythmias (74, 94). Similarly, Pgc-1β-/- transgenic mice reflecting mitochondrial dysfunction showed reduced atrial Cx protein expression (36). The latter finding may represent a direct consequence of ROS induced pathophysiology, although it may also be linked to the increased cardiac fibrosis which is seen in these mice, and discussed later in this review.

### Mitochondrial Dysfunction and Potassium Currents

Voltage gated potassium (K_V_) channels are regulated by cellular metabolism. K_V_ channels give rise to the transient outward K^+^ current (I_to_) underlying phase 1 repolarization, and the delayed rectifier K^+^ current (I_K_) underlying repolarization during phases 2 and 3 of the action potential (95). Electrophysiological studies on diabetic rats have demonstrated repolarization abnormalities resulting from downregulation K_V_ currents (96, 97). Experimentally, increased ROS has been shown to reduce I_to_ and I_K_ (including I_Kr_, I_Ks_ and I_Kur_) currents (74, 98, 99). This inhibition can be reversed through application of cellular antioxidant glutathione (74, 97, 100). ROS reduces K_V_ currents through reducing channel mRNA and protein expression levels (74, 99, 101). Peroxisome proliferator-activated receptor α (PPARα) upregulation during metabolic dysfunction has specifically been associated with reduced transcription of K_V_ channels (102). Additionally, ROS modulates K_V_ channel function by altering their phosphorylation status particularly acting through PKC and PKA (74, 103). Reduced K_V_ currents results in repolarization abnormalities resulting in prolonged action potential duration (APD) promoting EAD arrhythmic triggers (9, 95). Furthermore, altered APD/ effective refractory period (ERP) ratios result in spatiotemporal heterogeneity in activation and repolarization hence furnishing an arrhythmic substrate for re-entry arrhythmias (9, 81, 95).

Another group of K^+^ channels conduct an inwardly rectifying K^+^ current (K_ir_). These include sarcolemmal ATP-sensitive K^+^ channels. (sarcK_ATP_) predominantly formed by Kir6.2 and SUR2A and are important in the electrophysiological response to stresses such as ischemia (104). These are activated by a reduced ATP/ADP ratio during metabolic stress (105). The high density of sarcK_ATP_ channels means that only 1% of those channels need to open to significantly shorten the APD and hence the ERP and action potential wavelength (9, 106). Furthermore, opening of a large number of channels drives the membrane potential toward E_K_ causing the cardiomyocyte to become hyperpolarized and unexcitable (107). Thus, opening of sarcK_ATP_ channels generates a “current sink” which can slow or block action potential propagation (108). Together, these changes promote re-entrant arrhythmias (9, 106, 108, 109).

## Mitochondrial Dysfunction and Disrupted Calcium Homeostasis

With a 10,000-fold transmembrane gradient, Ca^2+^ is the most tightly regulated intracellular ion being utilized virtually ubiquitously in cellular signaling pathways (110, 111). Cardiomyocyte Ca^2+^ homeostasis is heavily influenced by cellular metabolism with increased ROS levels increasing cytosolic Ca^2+^ concentration ([Ca^2+^]_i_) (74, 112). These findings are summarized in Figure 1. The addition of H_2_O_2_ generating ROS in guinea pig ventricular myocytes resulted in increased current through the L-type voltage gated Ca^2+^ channels (I_CaL_) and hence significantly increased [Ca^2+^]_i_ (113). This, however, was reversed by application of the mitochondrial inhibitor myxothiazol or the L-type channel inhibitor nisoldipine (113). CAMKII activated by ROS has been shown to increase I_CaL_ via phosphorylation of the Ca_V_1.2 subunit (114) and similar accentuating effects on I_CaL_ were induced by oxidized LDL in rat ventricular cardiomyocytes (115). Furthermore, L-type channel appear to undergo direct redox modification and glutathionylation at cysteine residues in the alpha interacting domain (116, 117). Interestingly, the effect of ROS accentuating I_CaL_ has been challenged by other findings obtained under different experimental conditions which reported reduced I_CaL_ following oxidative stress (118).

In addition to sarcolemmal Ca^2+^ entry, ROS modulates intracellular Ca^2+^ handling proteins. Both canine and rat cardiomyocytes show increased opening of RyR2 in response to elevated ROS which triggers RyR2 Ca^2+^ sparks and accentuated Ca^2+^ efflux from the sarcoplasmic reticulum (119–121). Similarly, old rabbit hearts had more depolarized mitochondria membrane potential and increased rate of ROS production associated with increased RyR activity and Ca^2+^ leak which was accentuated under conditions of β-adrenergic stimulation (122). Treatment with antioxidant dithiothreitol reduced RyR-mediated SR Ca^2+^ leak to levels of young hearts highlighting the role of thiol-oxidation of RyR in underlying pathological SR Ca^2+^ release (122). This response also appears to depend on calmodulin as a functional mediator of ROS-triggered Ca^2+^ release (119). In contrast to RyR2, the sarco/endoplasmic reticulum Ca^2+^-ATPase (SERCA) activity is reduced in response to ROS (123, 124). SERCA inhibition by oxidative stress appears to arise through multiple mechanisms including reduced ATP supply (125), direct oxidation of thiol groups by ROS (123), and CAMKII-dependent phosphorylation (74, 126). Interestingly, adult rat ventricular myocytes expressing redox-insensitive SERCA where C674 is replaced by serine (C674S) decreased basal SR calcium content, attenuated the rise in mitochondrial Ca^2+^, and prevented cytochrome c release and apoptosis (127). Furthermore, beyond ROS generation, dysfunctional mitochondria contribute to disrupted Ca^2+^ homeostasis through reduced Ca^2+^ storage capacity. Mitochondria function as an important cellular Ca^2+^ store with Ca^2+^ ions entering the inner mitochondrial membrane via the mitochondrial Ca^2+^ uniporter (MCU) (128). However, under conditions of metabolic stress, mitochondrial Ca^2+^ handling is disrupted (101). This results in increased size and frequency of cytosolic Ca^2+^ transients resulting in arrhythmogenic Ca^2+^ alternans (129). For example, in rat ventricular myocytes, stress induced by thoracic aortic banding enhanced mitochondrial Ca^2+^ accumulation and hence disrupted global Ca^2+^ handling, increased spontaneous Ca^2+^ waves, shortened RyR refractoriness and decreased SR Ca^2+^ content (130). These effects were inhibited by MCU inhibitor Ru360 which normalized RyR oxidation state, improved intracellular Ca^2+^ homeostasis and reduced triggered activity (130). However, other studies have produced contradicting evidence (130–133). In rabbit atrial myocytes MCU inhibitor Ru360 increased the severity of Ca^2+^ alternans whereas stimulation of Ca^2+^ uptake was protective (133). In fact, diabetic cardiomyopathy has been associated with abnormal mitochondrial Ca^2+^ handling with altered MCU expression and reduced mitochondrial Ca^2+^ levels (134, 135). As such, the mechanisms by which mitochondrial dysfunction contributes to abnormal Ca^2+^ transients remain controversial with further experiments required to clarify this relationship particularly in the context of the pro-arrhythmic aging heart.

Intriguingly, mitochondrial Ca^2+^ overload itself contributes to mitochondrial dysfunction and ROS generation (136, 137). This perpetuates a positive feedback cycle of ROS-induced Ca^2+^ overload, Ca^2+^ -induced ROS generation, and ROS-induced ROS release (74, 138, 139). This occurs via multiple mechanisms. Firstly, Ca^2+^ stimulation of the Krebs cycle and the electron transport chain increasing electron leakage and ROS by-products (140, 141). Secondly, Ca^2+^-cardiolipin complexation disrupting mitochondrial lipid and protein arrangement causing proteins such as cytochrome c dislocation and inhibition of the electron transport chain and ROS production (142, 143). Thirdly, through activation of nitric oxide synthase generating NO radicals which itself has been shown to disrupt Ca^2+^ handling proteins (144, 145) but also to inhibit components of the respiratory chain and promote ROS production (146, 147).

Therefore, age-related mitochondrial dysfunction results in disrupted cellular Ca^2+^ handling causing elevated [Ca^2+^]_i_. The pro-arrhythmic consequences of elevated [Ca^2+^]_i_ are evident in CPVT hearts occurring due to mutations in cellular Ca^2+^ handling components, typically RyR2 or calsequestrin (148–150). This leads to potentially fatal ventricular arrhythmic episodes, often biventricular or polymorphic ventricular tachycardia and VF (151, 152). Interestingly, compared to aging mice where mitochondrial dysfunction disrupts multiple aspects of Ca^2+^ homeostasis, 129/Sv mice modeling CPVT demonstrated that altered function of only one of the Ca^2+^ handling proteins is sufficient to result in the proarrhythmic phenotype (150, 153).

Disrupted cardiomyocyte Ca^2+^ homeostasis develops a number of pro-arrhythmic pathways. Firstly, elevated [Ca^2+^]_i_ promotes the activity of the electrogenic NCX resulting in the generation of delayed after-depolarizations (DAD) which act as arrhythmic triggers (154, 155). As such, ROS causing cytosolic Ca^2+^ overload has been shown to stimulate NCX activity in guinea pig ventricular myocytes (112, 156). Secondly, dysregulation of Ca^2+^ handling allows pathological Ca^2+^ cycling which has been associated with APD alternans and spatiotemporal heterogeneities in repolarization (9, 157, 158). Thirdly, cytosolic Ca^2+^ interacts with surface membrane Na_V_1.5 and Cx channels causing reduced conduction velocity. Thus, Ca^2+^ regulates Na_V_1.5 and reduces I_Na_ through (1) directly binding to the EF hand motif, (2) associating with calmodulin and binding to the IQ domain, and (3) CAMKII-mediated phosphorylation (89, 90, 159). Inhibition of Cx function occurs through activating calcineurin-dependent Cx phosphorylation (160). Finally, increased cytosolic Ca^2+^, through increased ROS production, promotes tissue fibrosis which is associated with slowed conduction velocity (161).

## Mitochondrial Dysfunction and Cardiac Fibrosis

Aging is associated with increased cardiac fibrosis. Histological analysis of human hearts also demonstrates age-related progressive increase in collagen content and myocardial fibrosis (162, 163). Clinically, this is reflected in echocardiographic studies in both males and females which showed increased left ventricular wall thickness representing increased left ventricular hypertrophy (LVH) with age even in the absence of cardiovascular risk factors such as hypertension (5, 164). As such, age-related myocardial fibrosis has been shown to reduce ventricular elasticity, compromise left ventricular filling, and cause diastolic dysfunction (164, 165). Similarly, experimental mouse models also demonstrate increased collagen deposition in the aging myocardium (166). Transgenic premature aging (Polg^*m*/*m*^) mice show increased interstitial and subendocardial fibrosis along with greater amyloid deposition, vacuolization of cytoplasm and hyaline cytoplasmic change (5, 167).

Increased cardiac fibrosis with age reflects a disruption in the equilibrium of extracellular matrix (ECM) synthesis and degradation. ECM synthesis is stimulated by fibrogenic growth factors, such as transforming growth factor (TGF)-β which induce fibroblast production of matrix proteins and protease inhibitors such as tissue inhibitors of metalloproteinases (TIMPs) (168). However, ECM degradation is dependent on tumor necrosis factor (TNF)-α and interleukin (IL)-1β stimulating fibroblast production of matrix metalloproteinases (MMPs) (168). Hence, reduced MMP expression and inhibited ECM degradation appears to play a pivotal role in increased tissue fibrosis. As such, aging in murine models was associated with reduced MMP-1 and MMP-2 transcription and activity (169, 170).

With age, elevated ROS generation increases TGF-β and its downstream effector connective tissue growth factor (CTGF) (168). TGF-β in turn activates Smad2/3 signaling inducing fibroblast proliferation, differentiation into myofibroblasts, and the production of ECM components such as fibrillar collagen, fibronectin, and proteoglycans (168, 171). This is supported by studies in C57BL/6 mice which found increased cardiac fibrosis under conditions of immune dysregulation and tissue inflammation known to promote ROS production (172). Additionally, mice overexpressing catalase targeted to the mitochondria shoed reduced cardiomyocyte hypertrophy and significantly diminished cardiac fibrosis (167). Similarly, knock-out of SIRT3, which deacetylates the regulatory component of the mitochondrial permeability transition pore (mPTP), resulted in mitochondrial dysfunction, increased ROS production and accelerated signs of cardiac hypertrophy and fibrosis (173). Cardiac fibrosis also appears to be regulated by the renin-angiotensin system (RAS) signaling (174, 175). Angiotensin II (ANG II) activates the pro-fibrotic Smad2/3 signaling directly by acting on the ANG II type 1 receptor (AT1) and indirectly by promoting TGF-β production (168, 176). Furthermore, RAS has been shown to increase ROS levels through mechanisms including activation of NADPH oxidase (168, 177). ROS in turn have been found to promote the pro-fibrotic effects of ANG II which were suppressed through the application of antioxidants and AT1 antagonist losartan (177). Consistent with this, aged rat hearts demonstrate significantly increased angiotensin converting enzyme (ACE) and ANG II concentrations (167, 178, 179). Hence, mice carrying a gain-of-function mutation in the Ang II receptor type 1A developed early and progressive cardiac fibrosis (180).

Clinical studies strongly associate fibrosis with increased arrhythmic risk. For example, the origin of arrhythmia post-myocardial infarction is often mapped to the fibrotic border of the infarcted zone in patients undergoing ablation for recurrent VT (181). Similarly, most cases of AF are thought to originate from the atrial myocardial sleeve extending into the pulmonary veins. Histological analysis of pulmonary veins of patients with AF demonstrates increased myocardial content characterized by severe hypertrophy and fibrosis (182). Isolated Langendorff-perfused explanted human hearts with extensive infarction or dilated cardiomyopathy demonstrated increased vulnerability to triggering of VT due to cardiac fibrosis facilitating re-entry mechanisms (183, 184). Correspondingly, aged (24 months) Kunming mice had greater electrocardiographic abnormalities and inducibility of AF compared to young (2 months) mice which was associated to age-related increase in atrial fibrosis (185). Furthermore, transgenic Mkk4 knockout mice had dysregulated MMP function and upregulated TGF- β signaling causing increased susceptibility to atrial tachyarrhythmias (186). Importantly, these effects were more prominent in aged than young mice (186).

Interestingly, despite the primary biophysical defect of Na_V_1.5 haploinsufficiency being present from birth, BrS symptoms occur mainly in adulthood with mean age of SCD in BrS patients being 40 years (187, 188). The increased arrhythmogenicity later in age has thus been attributed to age-related structural changes primarily cardiac fibrosis (25, 188). Hence, old Scn5a^+/−^ BrS mice demonstrated reduced conduction velocity and increased myocardial fibrosis compared to young mice (189, 190).

Age-related cardiac fibrosis increases arrhythmic tendency through a variety of mechanisms. Firstly, fibrosis causes slowed cardiac conduction velocity (82). Fibrosis creates strands of cardiomyocytes which are electrically isolated from each other by collagenous septa (191). Thus, this forces the action potential waves to follow a “zigzag” pattern, conducting circuitously from one strand to the other resulting in slowed conduction velocity (181, 192). Fibrosis also results in Cx-mediated cardiomyocyte-fibroblast coupling which increases cardiomyocyte membrane capacitance (C_m_) slowing down action potential propagation (193, 194). Additionally, fibrosis reduces myocyte-myocyte coupling by decreasing Cx expression and promoting their redistribution away from the intercalated discz and hence increasing axial resistance resulting in slowed conduction velocity (195–198). Secondly, spatial heterogeneity in cardiac fibrosis and hence in compromised Cx function and altered ionic currents, including reduced Na^+^ current density, promotes APD alternans and dispersions of refractoriness causing unidirectional conduction block arrhythmic substrate (199–202). Additionally, patchy or interstitial fibrosis creates cardiomyocyte strands that are electrically coupled to nonfibrotic regions. Hence, creating a situation that reflects a 1 dimensional cable entering a 3 dimensional syncytium at which the interface acts as a “current sink” generating a “current-sink mismatch” due to the unequal transfer of depolarizing charge (191). Thus, if charge transfer to the syncytium is insufficient to depolarize the syncytium then action potential propagation fails (203). On the other hand, conduction from the syncytium to the 1-dimensional cable will succeed as the source-to-sink ratio is reversed. Therefore, this establishes a unidirectional conduction block facilitating arrhythmic re-entry circuits (191, 204).

## Targeted Pharmacological Therapy

Elucidation of the mechanisms by which age-related mitochondrial dysfunction and ROS generation increases arrhythmic risk offers a number of potential anti-arrhythmic pharmacological targets. Some of these targeted therapies, differentiated from non-targeted antioxidant therapies, are highlighted in Table 1.

**Table 1:** Potential targeted therapeutics which may alleviate arrhythmogenic mitochondrial dysfunction.

**Mitochondria-targeted pharmacological therapy**	**Mechanism**	**Therapeutic effect**
**Antioxidants**		
TPP+ conjugated antioxidants	Highly lipophilic antioxidants conjugated to strongly positive cations accumulate in mitochondria	Reduce ROS production Reduce mitochondrial component oxidation Reduce ROS-induced apoptosis and necrosis
Szeto-Schiller peptides	Cationic tetrapeptides, accumulate in the inner mitochondrial membrane	Scavenge ROS Reduce lipid peroxidation Reduce Ca2+ induced mitochondrial swelling Reduce reperfusion injury
**Modifiers of mitochondrial biogenesis**		
SIRT1 activators	Upregulation of SIRT1 transcription	Increasing PGC-1α expression Antioxidant properties (see above) Reduced NF-κB activation Electrophysiological modifications: Inhibition of I_Na−L_, I_Ca−L_ Reduction of intracellular Ca^2+^ transients
Rapamycin	Inhibition of mTOR signaling	Reduced ROS production Reduced cardiac hypertrophy Normalization of age-related Ca^2+^ homeostasis disruption Increased SERCA expression Reduced RyR current amplitude Increased mitophagy

*PGC-1a, Peroxisome proliferator-activated receptor gamma coactivator 1-alpha; ROS, reactive oxygen species; RyR, ryanodine receptor; SERCA, sarco-/endoplasmic reticulum Ca^2+^-ATPase; TPP, triphenylalkylphosphonium ion*.

### Antioxidant Therapy

Since mechanisms of ionic current dysregulation, disrupted Ca^2+^ homeostasis, and increased fibrosis all occur downstream of mitochondrial dysfunction, then it is likely that targeting upstream mitochondrial dysfunction and ROS generation will result in significant anti-arrhythmic effects.

### Non-targeted Antioxidant Therapy

The first attempts to counteract oxidative damage in aging has been with the administration of non-targeted antioxidants such as vitamins E and C and β-carotene. While initial small studies indicated some protective effects of non-targeted antioxidants on cardiac function, meta-analysis of larger clinical randomized controlled trials collectively involving tens of thousands of patients found no significant positive effects of non-targeted antioxidants on cardiovascular health or overall mortality (205–207). This may be due to the types of antioxidants investigated by clinical studies. For example, vitamin E has been shown to have pro-oxidant effects (208). Endogenous non-targeted antioxidant enzymes such as superoxide dismutase and catalase which were used in experiments to support the use of antioxidant therapy are not feasible in a clinical setting due to their size, rapid degradation, and potential antigenicity (5).

The failure of non-targeted antioxidants in clinical studies coupled to experimental findings that the source of ROS in aging arises primarily from the mitochondria has motivated the development of mitochondria-targeted antioxidants.

### Triphenylalkylphosphonium Ion (TPP^+^) Conjugated Antioxidants

The highly negative mitochondrial membrane potential (150-180 mV) has been utilized to target molecules to the mitochondria. Thus, coupling lipophilic antioxidants to strongly positive cations such as TPP^+^ increases accumulation in the mitochondria by 100- to 1000-fold compared to the cytosol (5). Such antioxidants include coenzyme Q (MitoQ), plastoquinone (SkQ1), and piperidine nitroxide in combination with triphenylphosphonium chloride (MitoTEMPO) (209, 210). Experimental studies found that they significantly reduce ROS generation, oxidation of mitochondrial components such as cardiolipin, ROS-induced apoptosis and necrosis, and prolonged lifespan of the fungus Podospora anserina, the crustacean Ceriodaphnia affinis, Drosophila, and mice models (210–212). Additionally, SkQ1 inhibited development of age-related conditions including retinopathy and osteoporosis in mammalian models of those conditions (210). In a rat model of H_2_O_2_- and ischemia/reperfusion-induced cardias arrhythmias, treatment with SkQ1 for 3 weeks abolished the steady heart arrhythmia (213). Furthermore, experiments in a guinea pig model of non-ischemic heart failure that recapitulates features of prolonged QT interval and high incidence of spontaneous arrhythmic SCD, MitoTEMPO normalized cellular ROS levels, avoided and reversed heart failure, and prevented SCD by decreasing dispersion of repolarization and ventricular arrhythmias (214). So far, clinical trials are yet to investigate the anti-arrhythmic effects of TPP^+^ conjugated antioxidants on human patients.

Limitations of TPP^+^ conjugated antioxidants include their reliance on the mitochondrial membrane potential gradient. This gradient is disrupted with mitochondrial dysfunction in aging, as well as a direct effect of the antioxidants at high concentrations hence limiting their uptake and effectiveness (5, 211, 212). Additionally, at higher micromolar concentrations, these molecules appear to show pro-oxidant rather than antioxidant effects (212). It is thus important to clarify the window between anti- and pro-oxidant concentrations before proceeding to clinical trials.

### Szeto-Schiller (SS) Peptides

SS peptides are synthetic aromatic-cationic tetrapeptides that selectively target and concentrate in the inner mitochondrial membrane (215, 216). Hence, *in vitro* experiments have shown that SS peptides scavenge ROS including hydrogen peroxide, hydroxyl radical, and peroxynitrite (215, 217). As such, they prevent lipid peroxidation as well as Ca^2+^-mediated mitochondrial swelling or reperfusion injury by inhibiting mitochondrial permeability transition and cytochrome c release (215, 216, 218, 219). In mouse models of ANG II-induced cardiomyopathy and Gαq-overexpression induced heart failure, SS peptide administration prevented mitochondrial dysfunction and ROS generation, downregulated pro-oxidative pathways, and reduced cardiac hypertrophy and fibrosis (220). Similarly, in a rat model of ischemia-reperfusion injury, SS peptides significantly reduced myocardial lipid peroxidation and infarct size as well as reducing the frequency and severity of cardiac arrhythmias (221).

A significant advantage of SS peptides over MitoQ and SkQ1, is that SS peptides do not depend on the mitochondrial membrane potential gradient for accumulation in the mitochondria as they have been shown to concentrate in dysfunctional depolarized mitochondria (5, 215). Additionally, unlike most oligopeptides, SS peptides are water soluble and have good transcellular permeability (215, 222).

### Targeting Mitochondrial Biogenesis

#### SIRT1 Activators (Caloric Restriction Mimetics)

Caloric restriction (CR) has been identified as one of the most potent interventions to improve health and slow down aging (223). Though the beneficial effects of CR are likely multifactorial, the sirtuin family of NAD^+^-dependent histone deacetylases, of which the predominant mammalian isoform is SIRT1, appear to be responsible for a large number of those beneficial effects (224, 225). SIRT1 acts through multiple pathways to regulate inflammatory responses, cellular senescence and its associated secretory phenotype, telomere attrition, and DNA damage responses (161, 226). As such, aging and its related mitochondrial dysfunction and ROS production are associated with decreased SIRT1 expression and activity (227). Thus, expression of SIRT1 was induced in rats undergoing CR and in human cells exposed to serum from CR rats, and in turn SIRT1 deacetylated the DNA repair factor Ku70 and sequestered the proapoptotic factor Bax away from mitochondria (224, 227). In mice, gain-of-function mutation of SIRT1 improved endothelial function through activating endothelial NO synthase (eNOS), preventing ROS production, inhibiting NF-κB signaling by deacetylating RelA/p65, and reducing the inflammatory response (228). Similarly, other experiments replicated the positive effects of SIRT1 activation including enhanced mitochondrial biogenesis by inducing eNOS expression (229). Therefore, it is expected that compounds capable of activating SIRT1 will recapitulate the protective anti-aging effects of caloric restriction and hence prolong life and improve cardiovascular health including reduced arrhythmic risk. Resveratrol is one such compound being investigated. Its presence in red wine is thought to account for the cardiovascular protective effects of red wine drinking particularly in southern France (230). Resveratrol induced similar transcription profiles as SIRT1 and CR and promoted the same protective effects in heart, skeletal muscle and brain tissue in mice where it also prolonged lifespan and prevented age-related cardiac dysfunction (231).

One of the main mechanisms through which SIRT1 acts is through stimulating PGC-1α expression which acts as an important regulator of mitochondria bioenergetics (232, 233). In rats, resveratrol demonstrated significant antioxidant properties in cultured aortic segments and endothelial cells through reducing ROS production and damage by reducing H_2_O_2_ levels and H_2_O_2_-mediated apoptosis, preventing UV-induced DNA damage, as well as increasing expression of antioxidant enzymes glutathione peroxidase, catalase, and heme oxygenase-1 (230). Similar antioxidant effects were reported in experiments using guinea pigs (234). Furthermore, it inhibited NF-κB activation and reduced vascular tissue inflammation (235). As such, it has been shown to block age-related cardiac hypertrophy and fibrosis in animal models (173, 236, 237). Interestingly, resveratrol has been suggested to normalize intracellular Ca^2+^ in a murine model of chronic diabetes through increasing SERCA2a expression (238, 239). Moreover, resveratrol exerts its antiarrhythmic effects on cardiac electrophysiology through regulating a number of ionic currents including inhibition of I_Na−L_, inhibition of I_CaL_ and reduction in the amplitude of intracellular Ca^2+^ transients, (232, 237, 240, 241). Intriguingly, resveratrol effects on repolarization currents appear more complex with studies finding contradictory changes, nonetheless, in all of those studies the change exerted antiarrhythmic effects (232, 233, 241–243). Additionally, resveratrol promotes the inotropic effect of sympathetic stimulation, without enhancing their proarrhythmic effects and hence evading sinoatrial tachycardia (244).

Therefore, in a rat model where ventricular arrhythmias are enhanced via ischemia-reperfusion, application of resveratrol significantly reduced the severity of ventricular arrhythmia and mortality rate (245, 246). Similarly, in a rabbit model of heart failure, inducibility of atrial fibrillation was markedly reduced by treatment with resveratrol (237). These antiarrhythmic properties have been demonstrated under a number of different experimental models (232, 233, 240, 243) confirming the potential of resveratrol to act as an effective cardioprotective antiarrhythmic agent. While significant clinical data regarding the protective effects of resveratrol, particularly its antiarrhythmic potential, are yet to be obtained, initial clinical trials focusing pharmacokinetics and metabolism of resveratrol have found it to be safe and reasonably well-tolerated at doses of up to 5 g/day (247).

#### Rapamycin and mTOR

In addition, mammalian target of rapamycin (mTOR) is an important component of nutrient signaling pathways implicated in the aging process (248). mTOR is a protein kinase that forms the core of two protein complexes, mTOR complex 1 and mTOR complex 2, which play an important role in aging through regulation of a variety of cellular pathways controlling cell growth and proliferation (249). Of those, complex 1 appears to be more important in cardiac aging accelerating ribosomal synthesis and cap-dependent translation through phosphorylation of p70S6K (S6K1) and 4E binding protein 1, respectively (5, 249). mTOR signaling is increased with age reflecting its role in the aging mechanism but is normalized with caloric restriction in mice (250).

Inhibition of mTOR signaling through rapamycin has been shown to prolong lifespan in numerous animal models including mice (251). In a murine model of load-induced cardiac hypertrophy via aortic constriction, rapamycin application suppressed S6K1 levels and prevented cardiac hypertrophy (252). Furthermore, application of rapamycin following established cardiac fibrosis improved ventricular function and reversed cardiac fibrosis (253, 254). Similar results were replicated clinically where patients who received rapamycin following cardiac transplant had reduced cardiac hypertrophy and improved cardiac function (255). Rapamycin has also been shown to normalize age-related disruption in ion homeostasis particularly of Ca^2+^. As such, rapamycin increased SERCA expression, and reduced RyR current amplitude, elevation in [Ca^2+^]_i_ and activation of downstream Ca^2+^ pathways such as mitogen-activated protein (MAP) kinases (253, 256, 257). Mitochondrial ROS production and pro-arrhythmic disturbances in Ca^2+^ homeostasis are also caused by age-related decrease in mitochondrial autophagy (mitophagy) (258–260). Autophagy is negatively regulated by mTOR. Hence enhancing autophagy via Torin1 potent mTOR inhibitor in aged rabbit hearts reduced the rate of ROS production and restored both the depolarized mitochondrial membrane potential and defective Ca^2+^ handling (261). Therefore, rapamycin pharmacological inhibition of mTOR may offer feasible anti-aging and hence anti-arrhythmic therapy. However, the anti-arrhythmic effects are yet to be explored by laboratory and clinical studies.

## Conclusion

Aging is a cardinal risk factor for arrhythmic incidence in the general population and in individuals with inherited channelopathies. Aging is closely related to mitochondrial dysfunction which promotes arrhythmogenesis whereby it increases arrhythmic triggers and substrates via modifying sodium (Na_V_1.5) and potassium (K_V_, sarcK_ATP_) ion channels, gap junctions, Ca^2+^ homeostasis (Ca_V_1.2, SERCA, RyR), and tissue fibrosis. Hence, stratification using “mitochondrial health” as a marker of arrhythmic risk such as through the utilization of metabolomics to analyze biopsy samples allows identification of vulnerable patients amenable to pharmacological therapy. As such, a number of exciting pharmacological therapies targeting mitochondrial dysfunction have been discussed including targeted antioxidants (TPP^+^-conjugated antioxidants and Szeto-Schiller peptides), SIRT1 activators (resveratrol), and mTOR inhibitors (rapamycin).

## Author Contributions

KS: conceptualization. KS and IF: writing—original draft preparation, writing—review, and editing. Both authors have read and agreed to the published version of the manuscript.

## Conflict of Interest

The authors declare that the research was conducted in the absence of any commercial or financial relationships that could be construed as a potential conflict of interest.
